# Supplementary figures and images for: Open educational resources for distributed hands-on teaching in molecular biology
Source: PLoS One. 2025 Aug 5;20(8):e0327975. doi: 10.1371/journal.pone.0327975 (PMC12324123; doi:10.1371/journal.pone.0327975)

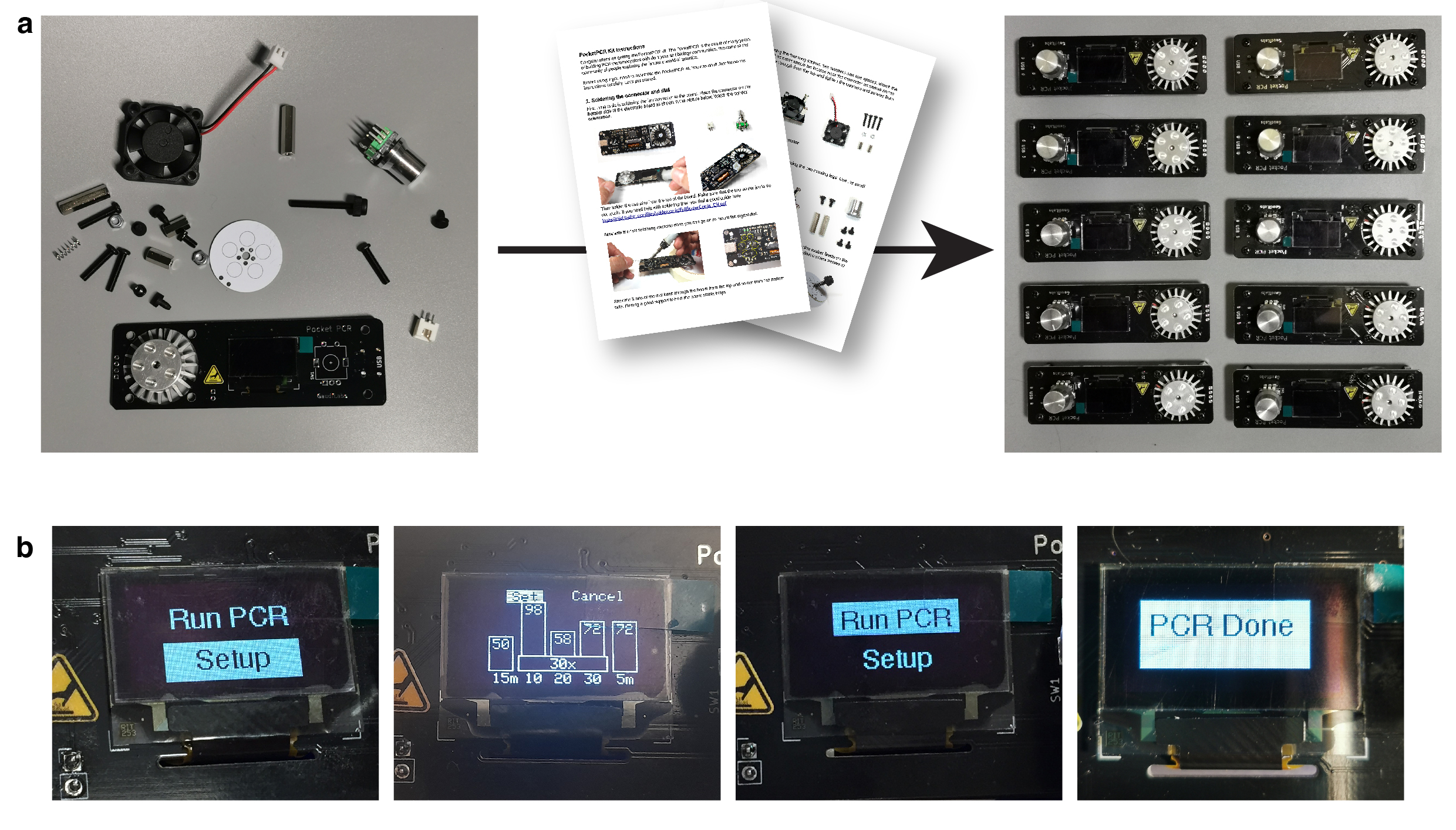

Supplement: S1 Fig — Clear instructions are provided by GaudiLabs. b) Sequential steps showing thermocycling setup. The main knob is rotated to choose values and pushed to confirm selections. (JPG) [file pone.0327975.s001.jpg]

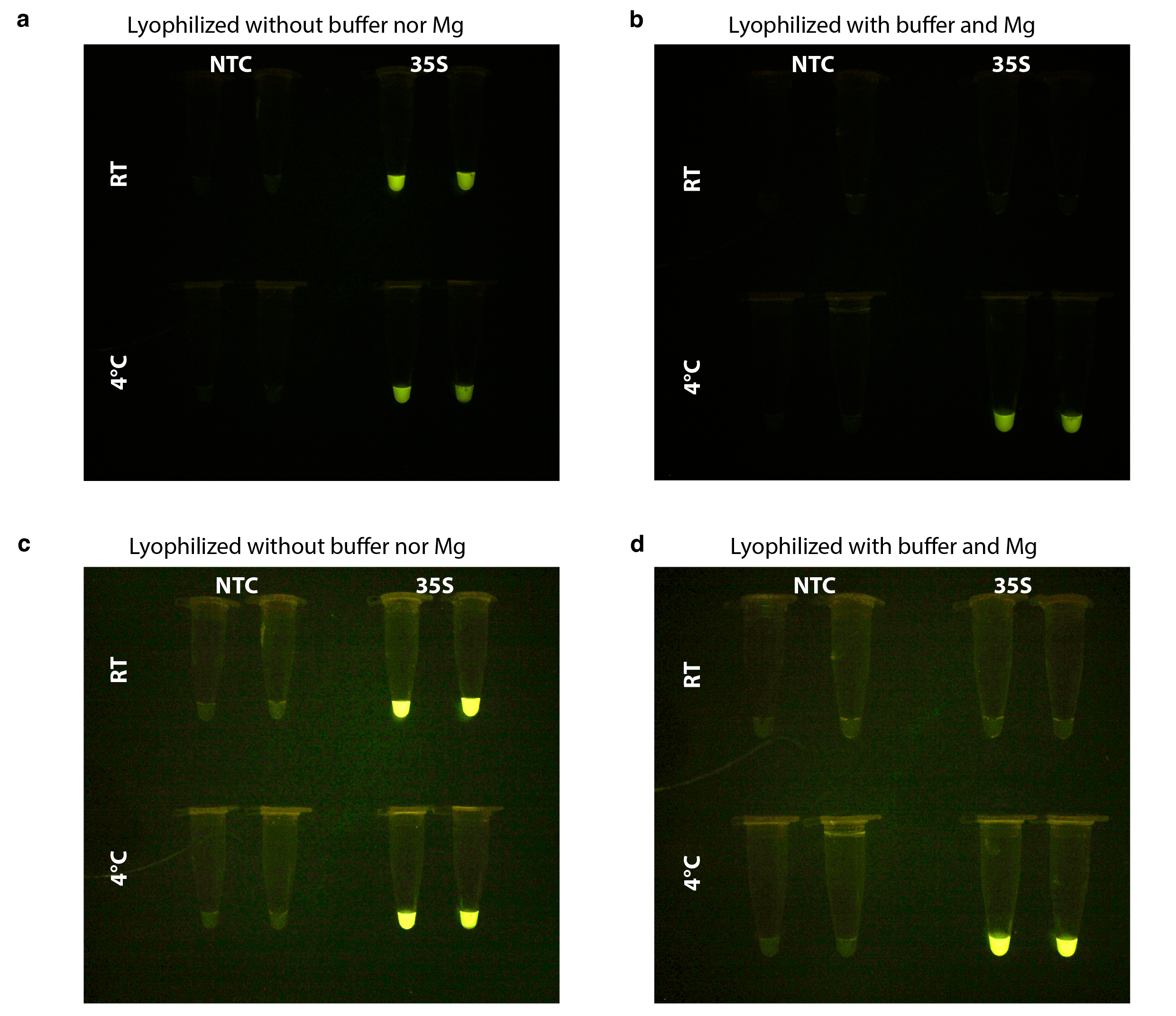

Supplement: S2 Fig — a) and c) Reactions lyophilized without buffer nor magnesium, rehydrated after two-month storage at room temperature (top) or 4°C (bottom). b) and d) Reactions lyophilized with buffer and magnesium, rehydrated after two-month storage at room temperature (top) or 4°C (bottom). Panel c and d are images with overexposure to show the negative tubes. (JPG) [file pone.0327975.s002.jpg]

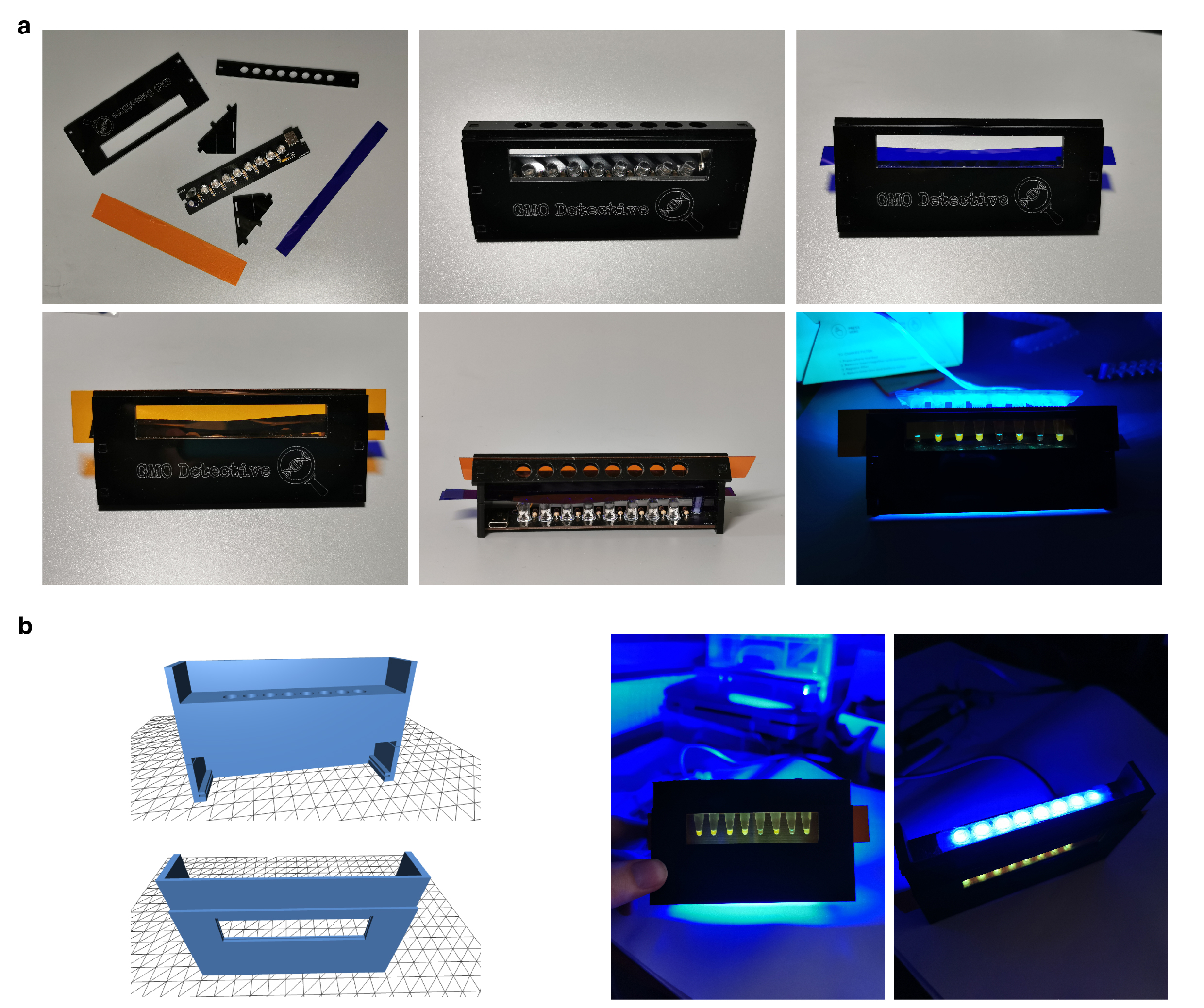

Supplement: S3 Fig — a) Acrylic version of the GMO Detective device. b) 3D printed version designed in openSCAD to accommodate the same filters and PCB used in the original device. All editable files available in https://github.com/MakerLabCRI/GMODetective-Detector/blob/main/3D%20Model/STL/GMOdetective3D.stl (JPG) [file pone.0327975.s003.jpg]

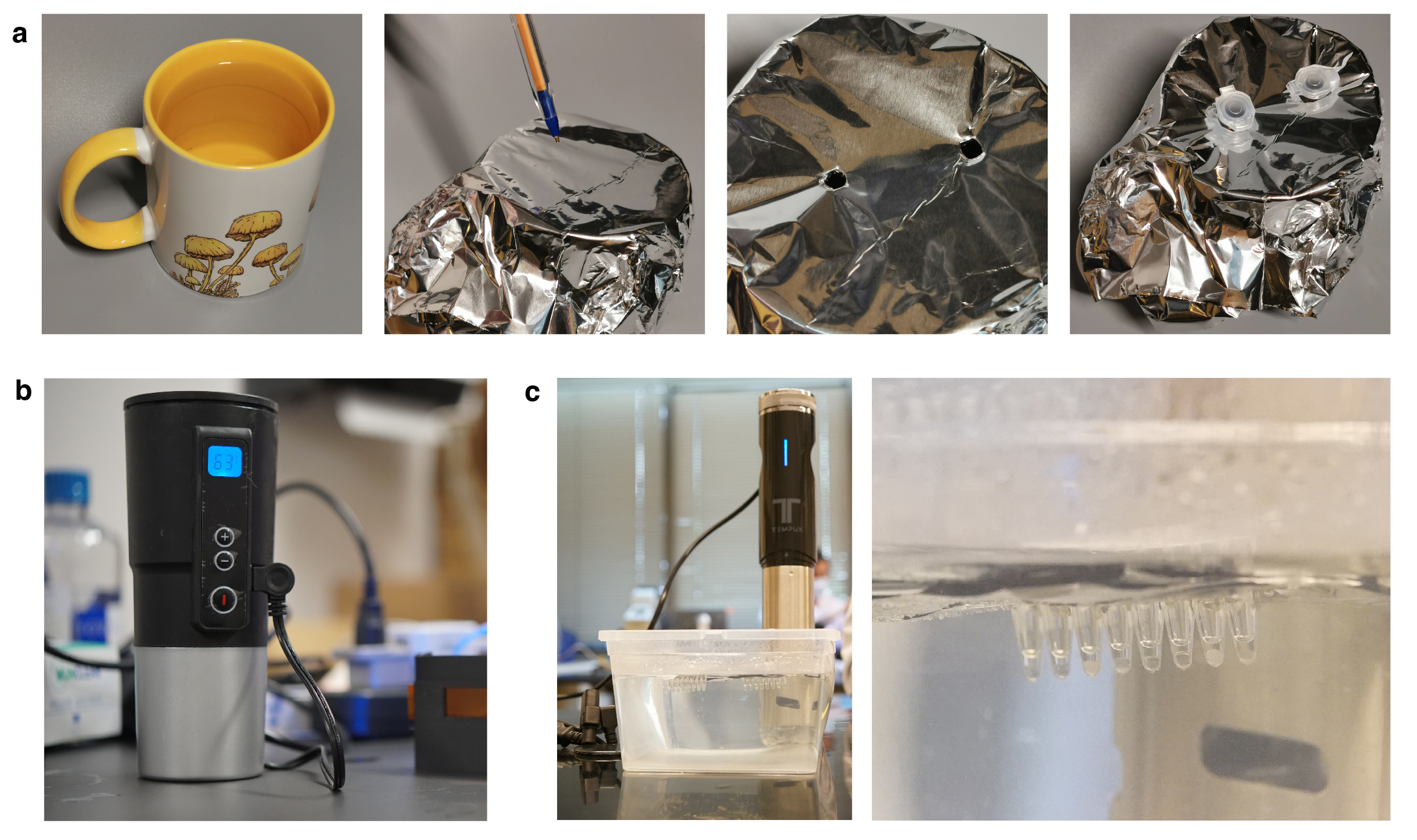

Supplement: S4 Fig — a) Step-by-step preparation of 100°C water incubation using aluminum foil for the initial 5 min heat treatment. b) Electronic coffee cup used for the 65°C incubation step. c) Sous vide heating device in a water bath used for the 65°C incubation step. (JPG) [file pone.0327975.s004.jpg]

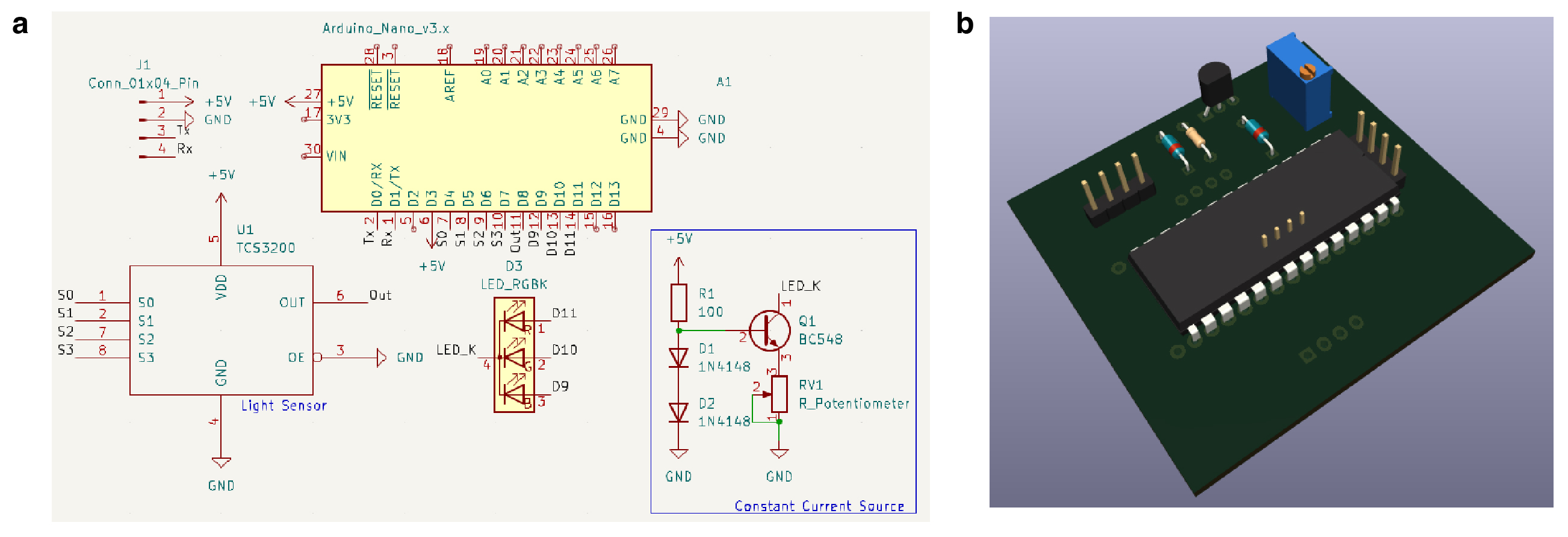

Supplement: S5 Fig — a) Schematic of the Arduino shield for the colorimeter reGOSH. b) 3D render of the colorimeter PCB. More details in https://gitlab.fcen.uncu.edu.ar/regosh/colorimetro-regosh (JPG) [file pone.0327975.s005.jpg]

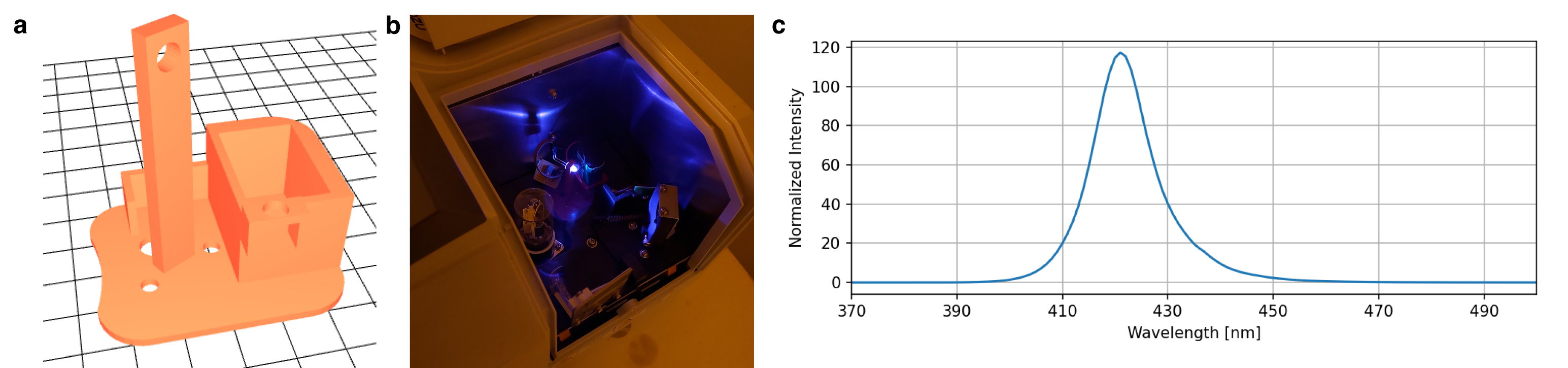

Supplement: S6 Fig — a) Schematic representation of the 3D printed LED adapter for the spectrometer’s light source compartment. b) UV LED placed inside the spectrometer. c) Relative intensity spectrum of the UV LED. (TIF) [file pone.0327975.s006.tif]

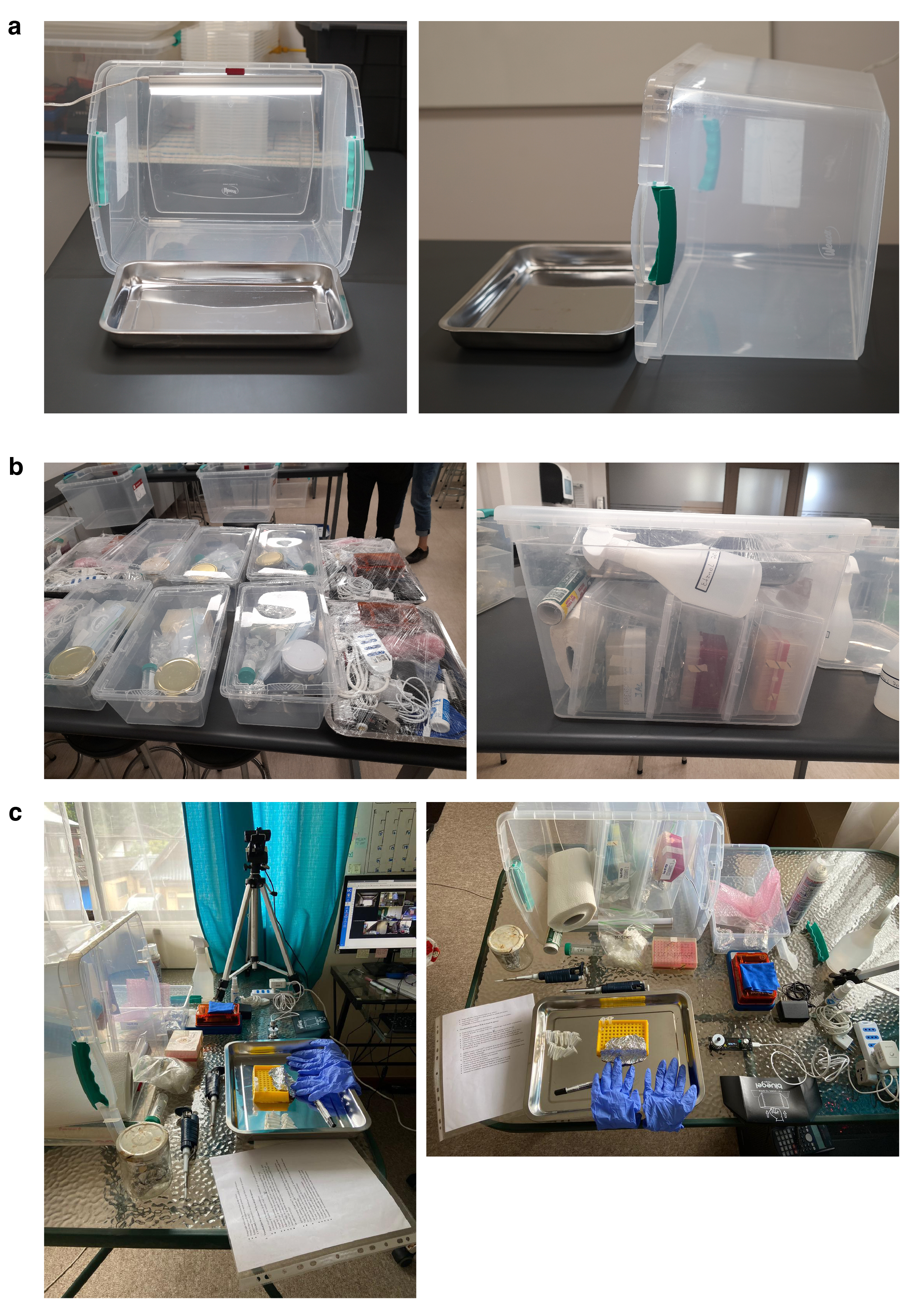

Supplement: S7 Fig — A light metallic tray was provided to run experiments at home. The box contained three smaller plastic boxes, one per student, which can be used as a water bath for LAMP experiments. b) organization of the three smaller boxes inside the lab-in-a-box. c) The correct employment of the box was monitored by Zoom video calls (image courtesy of Josefina Lara). (JPG) [file pone.0327975.s007.jpg]
