# Supplementary material for: Open educational resources for distributed hands-on teaching in molecular biology
Source: PLoS One. 2025 Aug 5;20(8):e0327975. doi: 10.1371/journal.pone.0327975 (PMC12324123; doi:10.1371/journal.pone.0327975)
Supplement: S1 Appendix — Detailed modifications to the original GMO Detective protocol, including custom primer sets, QUASR probes, and comparison of LAMP reactions using different fluorescence labels. (PDF) [file pone.0327975.s008.pdf]

S3 Appendix: homemade GMO Detective

Based on the original GMO detective designed, produced and shipped from France [1], we made some modifications to primers as listed below:

Original 35S primer set

Name	Sequence 5'to3'	Modification
35S_FIP	aggcatcttcaacgatggccttaaaggaaggtggctcctaca	
35S_BIP	tgccgacagtggtcccaaagttgaagacgtggttggaacg	5' FAM
35S_F3	tgcccagctatctgtcactt	
35S_B3	tcccttacgtcagtggagat	
35S_FLoop	tcctttatcgcaatgatg	
35S_BLoop	agcatcgtgggaaaagaag	
35S_BIPQ	ACTGTCGGCA	3' Black Hole Quencher®-1 Or Iowa Black® FQ

Original COX1 primer set

Name	Sequence 5'to3'	Modification
COX F3	tatgggagccgtttttgc	
COX B3	aactgctaagrgcattcc	5' FAM
COX FIP	atggatttgrcctaaagtttcagggcaggatttcactattgggt	
COX BIP	tgcatttcttagggctttcggatccrgcgtaagcatctg	
COX F-Loop	atgtccgaccaaagattttacc	
COX B-Loop	gtatgccacgtcgcattcc	
COX FIPQ	YCAAATCCAT	3' Black Hole Quencher®-1 orIowa Black® FQ

Modified 35S primer set (modifications labeled in yellow)

Name	Sequence 5’to3’	Modification
35SZ-F3	aagatgcctctgccgaca	
35SZ-B3	cagcgtgtcctctccaaat	
35SZ-FIP	acgtggttggaacgtcttcttcccaaagatggaccccca	
35SZ-BIP	atctccactgacgtaagggatgatagaggaagggtcttgcga	5’ FAM
35SZ-LF	tccacgatgctcctcgtg	
35SZ-LB	acgcacaatcccactatcct	
35SZ-BIP-Q	cagtggagat	3’ Iowa Black FQ

Modified COX1 primer set (modifications labeled in yellow)

Name	Sequence 5’to3’	Modification
COX-F3	tatgggagccgtttttgc	
COX-B3	aactgctaagRgcattcc	
COX-FIP	atggatttgRcctaaagtttcagggcaggatttcactattgggt	5’ Texas Red
COX-BIP	tgcatttcttagggctttcggatccRgcgtaagcatctg	
COX-LF	atgtccgaccaaagattttacc	
COX-LB	gtatgccacgtcgcattcc	
COX-FIP-Q	YCAAATCCAT	3’ Iowa Black FQ

FAM: Fluorescein
Iowa Black FQ: Fluorescence quencher in the FAM range.

QUASR probe diagram used:

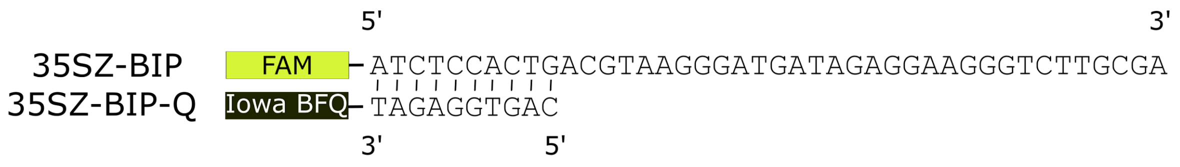

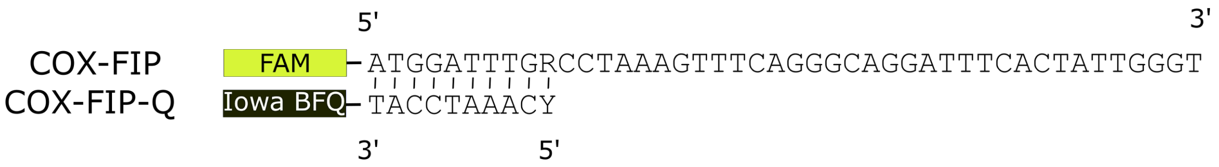

Annealing of LAMP primers for S.tuberosum mitochondrial cox1 gene [2]:

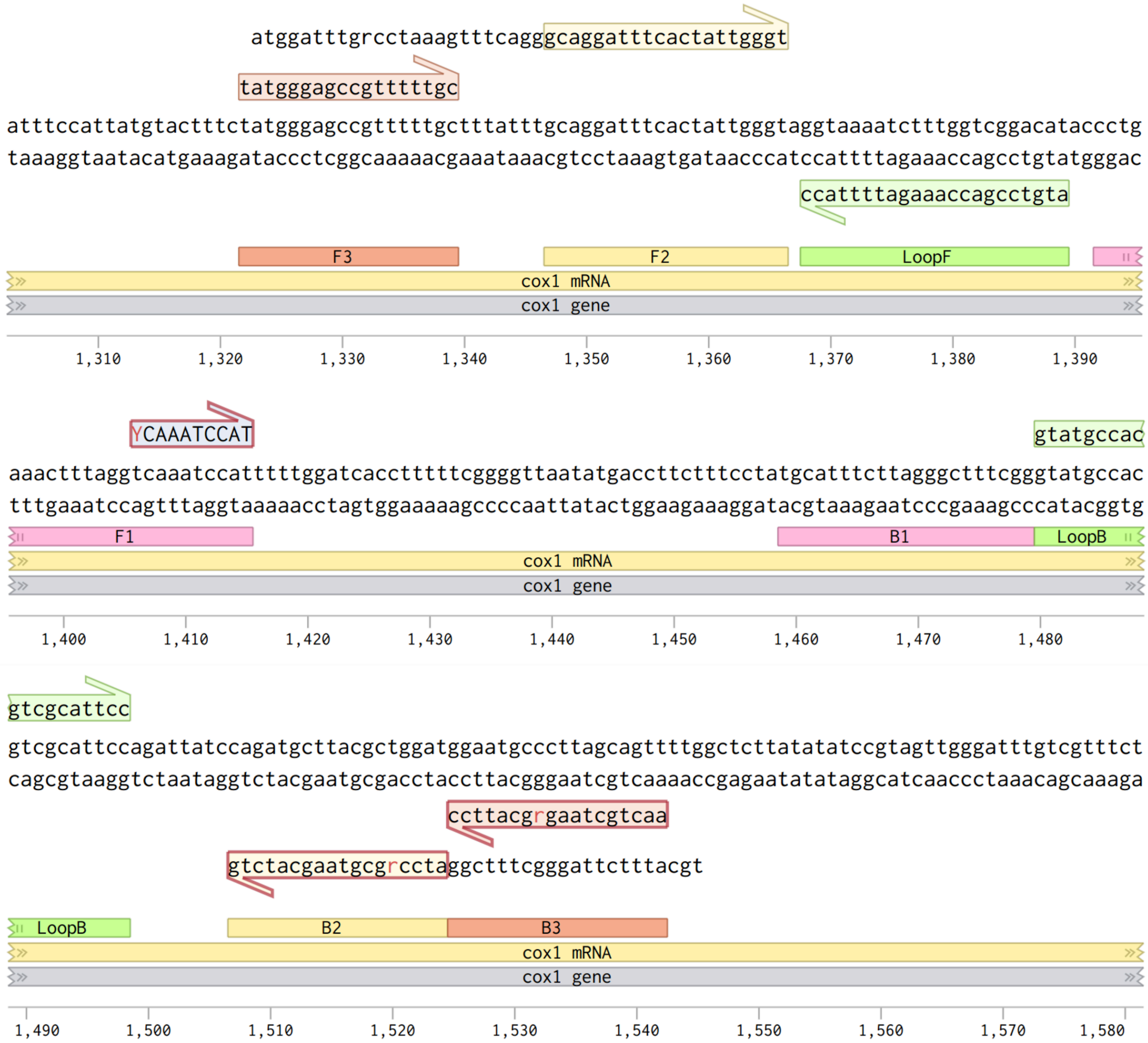

Annealing of LAMP primers for 35S CaMV [3]

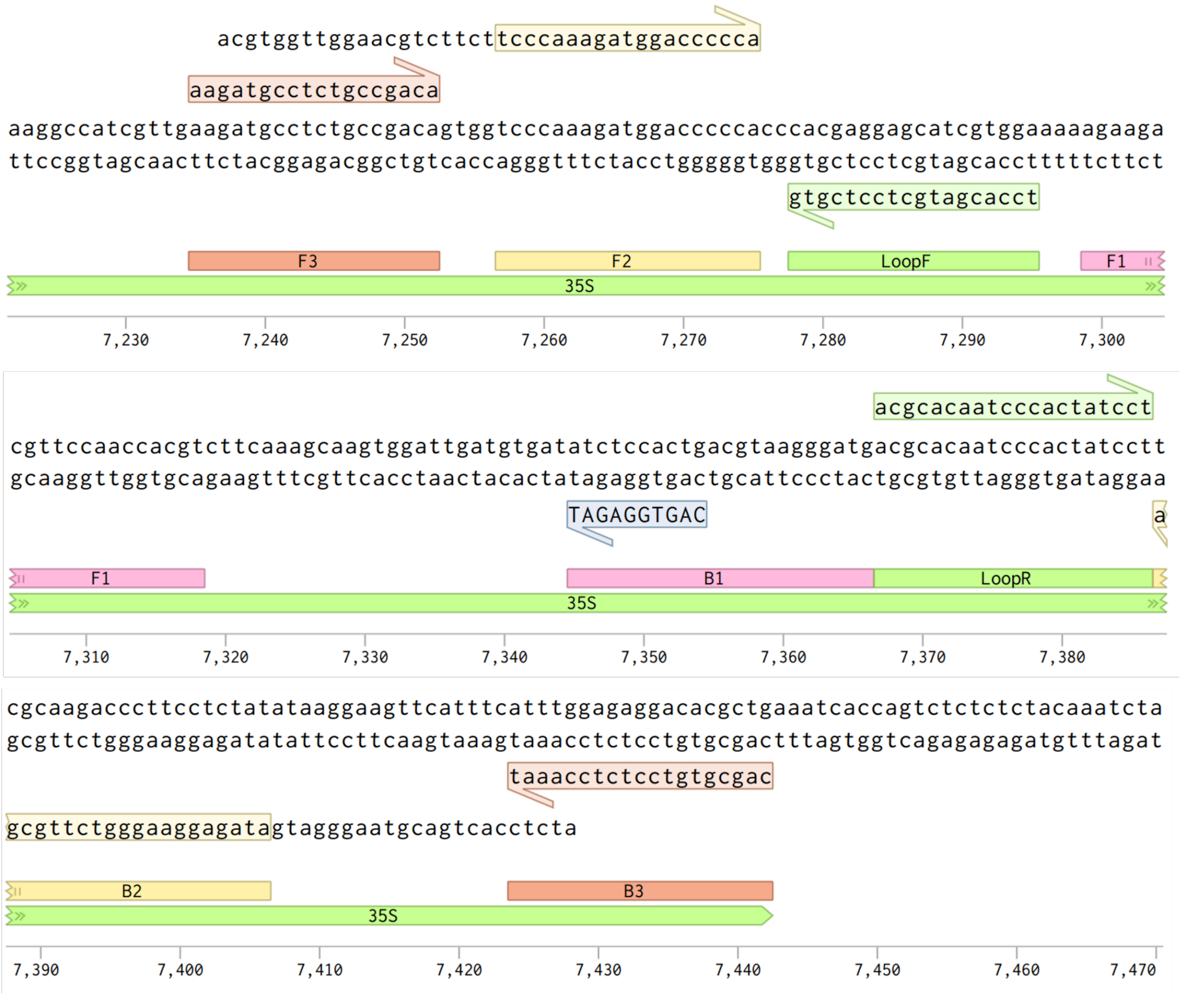

Primer quantities and purification methods at ordering

**Name**	**Sequence**	**Scale**	**Purification**		
COX-F3	tatgggagccgtttttgc	100nm	STD	100 nmole DNA oligo	Standard desalting
COX-B3	aactgctaagRgcattcc	100nm	STD	100 nmole DNA oligo	Standard desalting
COX-FIP	/5TexRd-XN/atggatttgR cctaaagtttcagggcaggattt cactattgggt	250nm	HPLC	250 nmole DNA oligo	**HPLC purification**

COX-BIP	tgcatttcttagggctttcggatccRgcgtaagcatctg	250nm	STD	250 nmole DNA oligo	Standard desalting
COX-LF	atgtccgaccaaagattttacc	100nm	STD	100 nmole DNA oligo	Standard desalting
COX-LB	gtatgccacgtcgcattcc	100nm	STD	100 nmole DNA oligo	Standard desalting
COX-FIP-Q	YCAAATCCAT/3IAbRQSp/	250nm	HPLC	250 nmole DNA oligo	**HPLC purification**
35SZ-F3	aagatgcctctgccgaca	100nm	STD	100 nmole DNA oligo	Standard desalting
35SZ-B3	cagcgtgtcctctccaaat	100nm	STD	100 nmole DNA oligo	Standard desalting
35SZ-FIP	acgtggttggaacgtcttcttcccaaagatggaccccca	250nm	STD	250 nmole DNA oligo	Standard desalting
35SZ-BIP	/56-FAM/atctccactgacgtaagggatgatagaggaagggtcttgcga	250nm	STD	250 nmole DNA oligo	Standard desalting
35SZ-LF	tccacgatgctcctcgtg	100nm	STD	100 nmole DNA oligo	Standard desalting
35SZ-LB	acgcacaatcccactatcct	100nm	STD	100 nmole DNA oligo	Standard desalting
35SZ-BIP-Q	C AGT GGA GAT/3IABkFQ/	250nm	HPLC	250 nmole DNA oligo	**HPLC purification**

Thermocycler

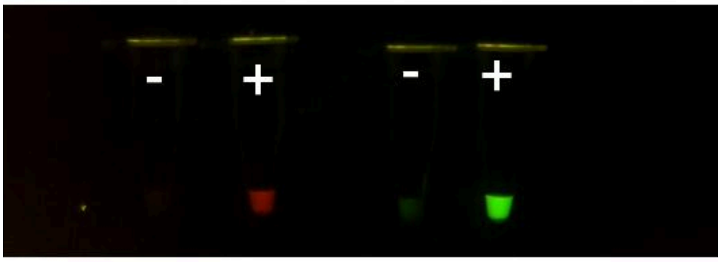

3D printer hot bed

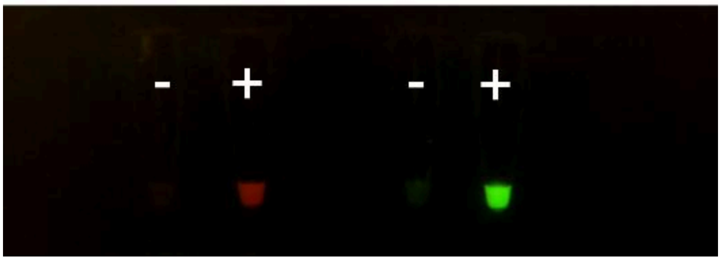

Comparison of Texas Red COX1 (left) and FAM 35S (right) QUASR LAMP reactions run on a thermocycler (top) and 3D printer hot bed (bottom). The thermocycler was run at at 63°C while the heated bed was run at 65°C. Homemade enzymes and buffers were used in both. The photo was taken after 60 min of incubation in a FluoPi [4](at 200 ISO, 760000 µs exposure time) .

1. [No title]. [cited 22 Feb 2024]. Available: https://gmodetective.com/wp-content/uploads/2018/10/GMO-Detective-Wetware-V1.1.pdf?189db0&189db0

2. COX1_X83206 · Benchling. [cited 22 Feb 2024]. Available: https://benchling.com/s/seq-jsZWJHYQhWAf3DYHAdUo

3. 35s_V00141 · Benchling. [cited 22 Feb 2024]. Available: https://benchling.com/s/seq-McxTSERY7eYYw9cOe7u9

4. Nuñez I, Matute T, Herrera R, Keymer J, Marzullo T, Rudge T, et al. Low cost and open source multi-fluorescence imaging system for teaching and research in biology and bioengineering. PLoS One. 2017;12: e0187163.
